# Neural Crest-Derived Mesenchymal Cells Require Wnt Signaling for Their Development and Drive Invagination of the Telencephalic Midline

**DOI:** 10.1371/journal.pone.0086025

**Published:** 2014-02-06

**Authors:** Youngshik Choe, Konstantinos S. Zarbalis, Samuel J. Pleasure

**Affiliations:** 1 Department of Neurology, University of California San Francisco, San Francisco, California, United States of America; 2 Program in Neuroscience, University of California San Francisco, San Francisco, California, United States of America; 3 Program in Developmental Stem Cell Biology, University of California San Francisco, San Francisco, California, United States of America; 4 Eli and Edythe Broad Center of Regeneration Medicine and Stem Cell Research and University of California San Francisco, San Francisco, California, United States of America; 5 Department of Pathology and Laboratory Medicine, University of California at Davis, Davis, California, United States of America; 6 Institute for Pediatric Regenerative Medicine, Shriners Hospitals for Children, Sacramento, California, United States of America; Institut Curie, France

## Abstract

Embryonic neural crest cells contribute to the development of the craniofacial mesenchyme, forebrain meninges and perivascular cells. In this study, we investigated the function of ß-catenin signaling in neural crest cells abutting the dorsal forebrain during development. In the absence of ß-catenin signaling, neural crest cells failed to expand in the interhemispheric region and produced ectopic smooth muscle cells instead of generating dermal and calvarial mesenchyme. In contrast, constitutive expression of stabilized ß-catenin in neural crest cells increased the number of mesenchymal lineage precursors suggesting that ß-catenin signaling is necessary for the expansion of neural crest-derived mesenchymal cells. Interestingly, the loss of neural crest-derived mesenchymal stem cells (MSCs) leads to failure of telencephalic midline invagination and causes ventricular system defects. This study shows that ß-catenin signaling is required for the switch of neural crest cells to MSCs and mediates the expansion of MSCs to drive the formation of mesenchymal structures of the head. Furthermore, loss of these structures causes striking defects in forebrain morphogenesis.

## Introduction

A unique feature of vertebrate neurulation is the delamination of neural crest progenitors from the dorsal neuroepithelium before and during neural tube formation. In mice, rostral neural crest cells detach from the closing neural tube by embryonic day (E)9.0, one day before the dorsomedial telencephalon invaginates to form the bilateral telencephalic vesicles, the prospective cerebral cortical hemispheres [1]. At E10.5, regional specification of the dorsomedial forebrain neuroepithelium divides areas of the hippocampus, the cortical hem, and the non-neural secretory choroid plexus, which extends into the lateral ventricle [2]. The secreted signaling factor Wnt3a is first expressed by the cortical hem at E10.5 in concordance with the invagination of the dorsal telencephalon [3].

In addition to the role Wnt signaling plays during the development of the central nervous system (CNS), this pathway is also known to exert important functions during induction and migration of neural crest cells. Wnt proteins activate an array of downstream target genes by stabilizing the intracellular signal transducer ß-catenin that binds Tcf family transcription factors in the nucleus and recruits co-activators. However, ß-catenin also binds to cadherins localized at adherence junctions contributing to the establishment of polarized epithelial tissues [4], [5]. Breakage of these junctions in epithelia outside the nervous system produces mesenchymal cells via a process termed epithelial-mesenchymal transition (EMT) [6], [7], [8]. Analysis of *Wnt1/Wnt3a* double mutants showed a profound loss of neural crest-derived structures, clearly demonstrating the critical role of Wnt signaling in the development of neural crest derivatives [9]. Interestingly, the neural crest-specific deletion of ß-catenin by using Wnt1-Cre mice showed both profound defects in neural crest-derived craniofacial structures and diminished neural precursor development in the forebrain [10], [11]. This raises the question of whether loss of Wnt signaling in head structures leads to separate mutant phenotypes in the cranial neural crest and forebrain, or whether there is a causal relationship between these two phenotypes.

Conditional inactivation of ß-catenin during mouse forebrain development using different Cre lines has thus far produced two distinct dorsal telencephalic phenotypes. First, mice with Emx1-Cre-dependent deletion of ß-catenin survive to adulthood without apparent neural crest defects while displaying diminished dorsomedial forebrain structures [12]. The dorsomedial structures properly invaginate forming bifurcated lateral ventricles. Contrastingly, Foxg1-Cre-mediated deletion of ß-catenin in both dorsal neuroepithelial and mesenchymal cells, results in severe loss of midline telencephalic structures, failure of midline invagination and associated craniofacial defects [13], [14], [15]. The marked difference in phenotypic alterations in these two mutant lines may stem from the loss of ß-catenin signaling in mesenchymal cells in *Foxg1-Cre;ß-catenin* mutants. Even though not clearly described in the existing literature, additional evidence for a correlation between cortical hem-mediated Wnt signaling and the failure of midline invagination through interstitial mesenchymal cells exists in several mouse mutants. For instance, the dorsomedial neuroepithelium of *Emx1/Emx2* compound mutants transforms into the roof plate with a diminished cortical hem and choroid plexus [16]. Loss of Emx1 and Emx2 expression is observed in *Gli3* (*Xt/Xt*) mutants [17], *Nestin-Cre;ß-catenin* mutants [18], *Gdf7-DTA* mutants [19], and ectopic *ShhN* expressing mutants [20]. The common feature in all of these mouse lines is diminished cortical hem-mediated Wnt signaling and incomplete midline invagination. From these seemingly separate mutant phenotypes, it is thus reasonable to investigate whether Wnt signaling to and from the mesenchyme and forebrain may regulate midline development.

Mesenchymal stem cells (MSCs) are among the most promising candidates for future cell-based therapeutic applications [21], [22]. Therapeutic MSCs are currently derived from newborn umbilical cord blood, adult bone marrow or adipose tissues. However, due to their mesodermal origin, these currently obtained MSCs may face limitations in their regenerative use for disorders of the forebrain and skull vault. Cranial neural crest cells are transient, highly migratory cells originating from the dorsal neuroepithelium before neural tube closure, migrating along the neuraxis, and contributing to a great variety of mesenchymal structures of the skull and forebrain vasculature [1]. Elements of the cranial skeleton such as the frontal bone develop by intramembraneous osteogenic condensation of mesenchymal cells derived from neural crest cells, which also contribute substantially to the meninges that cover the telencephalon [23], [24], [25]. In addition, neural crest cells produce non-neural cell types within the brain such as perivascular smooth muscle cells and pericytes [24]. The regenerative use of neural crest-derived MSCs may become very important for a range of disorders of the brain and skull and thus greater examination of the underlying developmental programs mediating their specification in the developing head is well warranted [26], [27], [28].

There are many questions remaining concerning the transition of cranial neural crest cells to mesenchymal progenitors forming the mesenchymal derivatives of head and face. Most importantly the molecules guiding conversion of neural crest cells to MSCs and their expansion along the rostrodorsal midline need to be further explored. We hypothesized that Wnts secreted from the cortical hem act as a proliferative signal on neural crest derived mesenchymal progenitor cells, which induces their expansion adjacent to the dorsomedial telencephalon and that this initial expansion of mesenchymal progenitor cells contributes to neural crest derived craniofacial structures and proper development of the forebrain. To address this question, we conditionally manipulated ß-catenin in late premigratory neural crest cells using Sox10-Cre mice [29]. Our results clearly demonstrate that Wnt/ß-catenin signaling plays a critical role in the development of neural crest-derived mesenchymal derivatives and that dorsomedial mesenchymal progenitors contribute to cortical midline invagination and lateral ventricle formation.

## Results

### Neural crest-derived mesenchymal stem cells in the dorsomedial interhemispheric region are Wnt responsive

**Figure 1:**
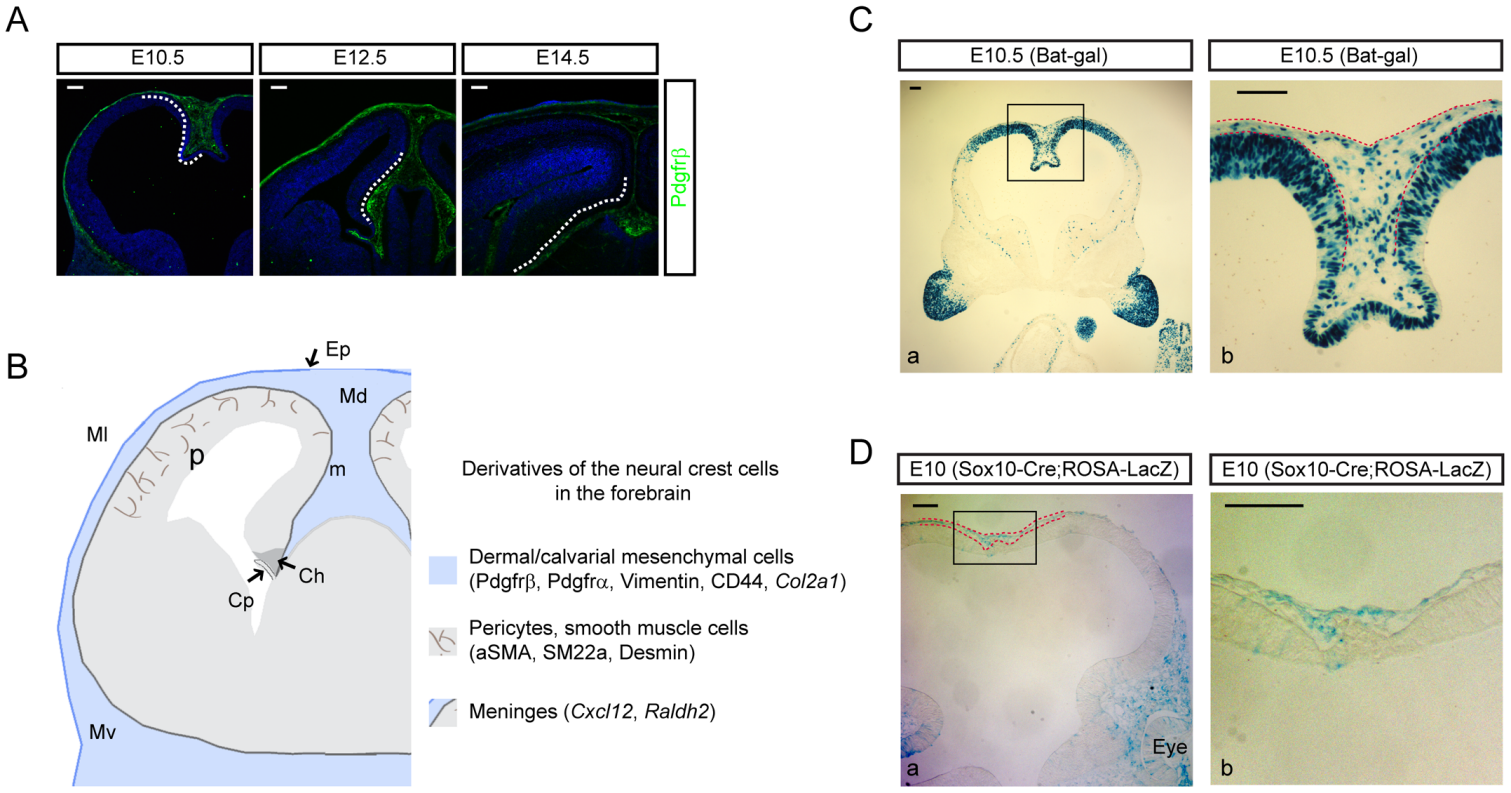
Wnt-responding mesenchymal cells expand in the dorsal interhemispheric region. **A**) Staining of Pdgfrß^+^ neural crest-derived mesenchymal cells from E10 to E14. Dashed lines highlight the dorsomedial mesenchymal cells, which expand at E10 and spread laterally at later ages. At E14, perivascular cells strongly express Pdgfrß. **B**) A diagram showing mesenchymal cells at the level of dorsoventral axis in the forebrain; the dorsal (Md), lateral (Ml), and ventral (Mv) mesenchymal cells. The derivatives of the neural crest cells are listed with markers used in this study. Ep = epidermis, Ch = cortical hem, Cp = choroid plexus, P = pericytes, m = meninges. **C**) X-gal staining of E10.5 Bat-gal transgenic embryos. A high power image of the boxed area of **C-a** is presented in **C-b**. X-gal^+^ mesenchymal cells are localized in the interhemispheric region (red dashed lines). **D**) X-gal staining of E10 *ROSA-lac*Z Cre reporter mice crossed with Sox10-Cre, a neural crest driver. A high magnification image of the boxed area in **D-a** is shown in **D-b**. Red dashed lines mark the area with mesenchymal cells. Scale bars = 100 µm.

Loss of canonical Wnt signaling in early-born neural crest cells leads to severe loss of forebrain structures, impaired neural crest lineages, and loss of neural crest-derived mesenchymal derivatives [9], [10], [11], [30]. In the rostral neural tube, Wnt3a expression starts in the dorsal neural epithelium and the cortical hem around E10.5 as the dorsal telencephalon invaginates to form the bifurcated hemispheric forebrain [3], [17]. To trace the developmental expansion of cranial neural crest-derived mesenchymal progenitors resident in the dorsal area, we used immunostaining for Pdgfrß, a well-described marker of this cell population [31]. Pdgfrß expression from E10.5–12.5 was seen widely in cells overlying the neuroepithelium but also concentrated at the dorsal midline as invagination proceeded (E10.5) and adjacent to the newly formed cortical hem (E12.5) (Fig. 1A). These Pdgfrß^+^ mesenchymal cells were densely distributed in the space between the telencephalon and the non-neural ectoderm. Two days later at E14.5, most of the mesenchymal cells were dispersed into the space between the epidermis and the cortex and Pdgfrß expression was reduced in the mesenchyme but remained unchanged in perivascular cells of the forebrain, the meninges, and epidermis (Fig. 1A, B). Since Pdgfrß^+^ mesenchymal cells reach their highest density in the dorsomedial region of the midline mesenchyme, apposed to the cortical hem, a source of Wnt secretion, we examined whether the cells were also responding to Wnt signaling at this stage. X-gal staining of Bat-gal transgenic embryos expressing the *lacZ* reporter under the control of Wnt-responsive elements revealed β-galactosidase^+^ (β-gal^+^) cells scattered along the mesenchyme of the dorsal cortical midline at E10.5 confirming Wnt responsiveness of these mesenchymal cells (Fig. 1C). This result raised the possibility that Wnt activation mediates proliferation of neural crest-derived MSCs in the dorsal interhemispheric region during invagination of the dorsal telencephalic midline. To test this idea we investigated suitable Cre driver lines specifically expressing Cre recombinase in neural crest-derived mesenchymal cells near the cortical hem during the period of dorsal neural tube invagination. We examined *Sox10-Cre;ROSA-lacZ* reporter mice, in which late premigratory neural crest cells are specifically recombined and found that this driver might be suitable [10], [29]. Indeed, X-gal staining in *Sox10-Cre;ROSA-lacZ* reporter mice at E9.5, confirmed β-gal expression both in interstitial neural crest cells of the rostrodorsal neural tube and emigrating dorsal neural crest cells (Fig. 1D).

### Activation of β-catenin in neural crest lineages expands mesenchymal cells

**Figure 2:**
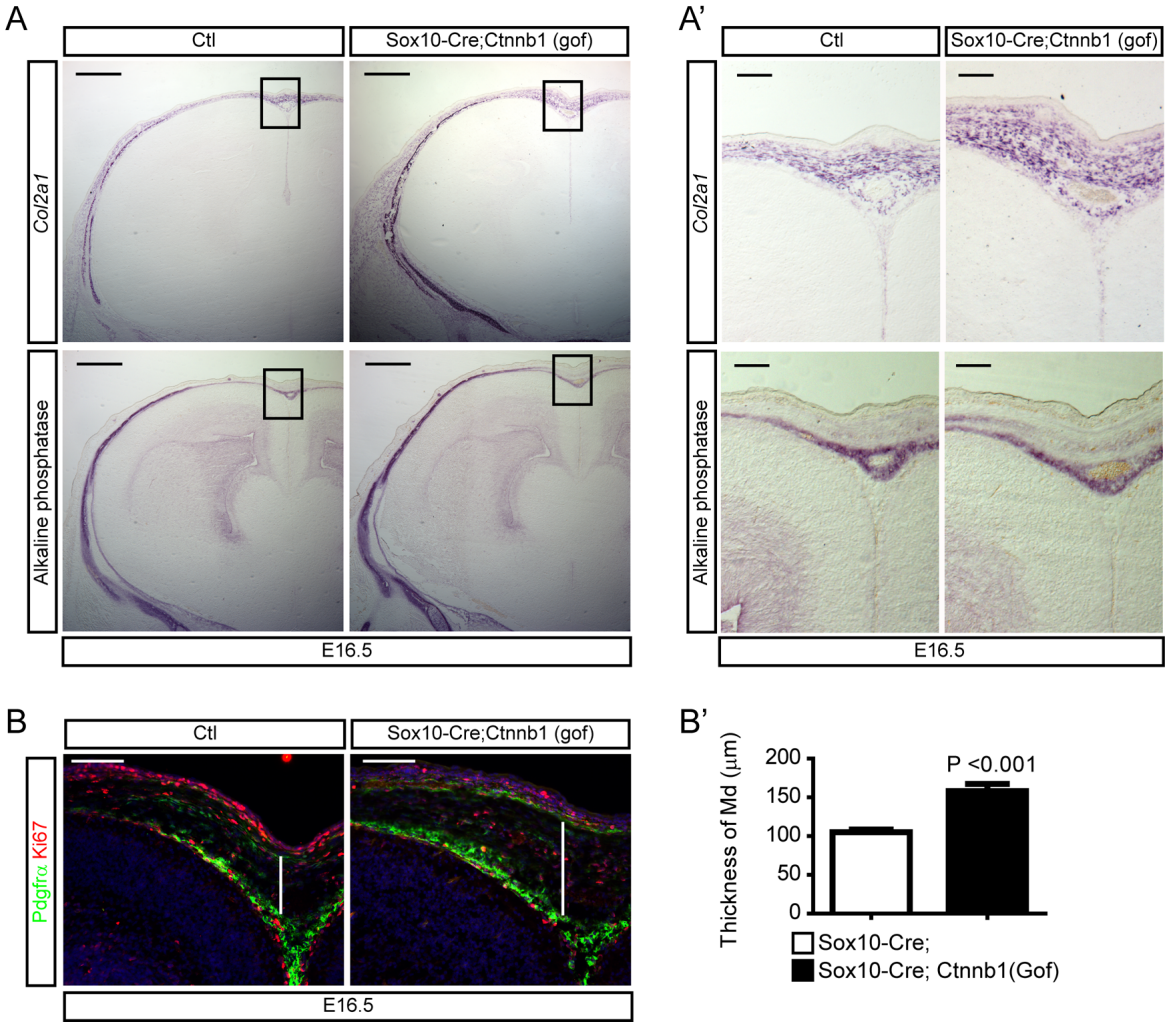
Expansion of mesenchymal cells by activation of β-catenin in neural crest cells. **A**) Mesenchymal cells of Sox10-Cre;Ctnnb1(gof) mutant at E16.5 marked by *Col2a1* expression (top) and alkaline phosphatase activities (bottom, osteoblasts) obtained from adjacent sections. Higher magnification images of the boxed areas are shown in **A′**. **B**) Mesenchymal cells were labeled for Pdgfrα and Ki67 to show the proliferating mesenchymal cells. **B′**) A graph shows thickness of dermal mesenchymal cells in the midline at E16.5 (white lines of **B**, n = 3). Error bar indicates SEM. Scale bars = 100 µm.

To understand the role of Wnt signaling in the development of the neural crest-derived MSCs we used mice expressing a constitutively active floxed version of ß-catenin [*Ctnnb1(gof)*] [32] and crossed these with *Sox10-Cre* mice. RNA *in situ* hybridization for *Col2a1*, a marker for chondrogenic mesenchymal cells [23], [33], revealed that *Col2a1^+^* mesenchymal progenitors were expanded dorsally and laterally in *Sox10-Cre;Ctnnb1(gof)* embryos at E16.5 (Fig. 2A, A′). At this developmental stage, Pdgfrß expression was restricted in perivascular cells while Pdgfrα expression was more ubiquitously seen in the mesenchyme (data not shown). Examining the dorsal midline in *Sox10-Cre;Ctnnb1(gof)* mice at higher resolution showed both an expanded population of Pdgfrα^+^ cells as well as increased number of proliferative Ki67^+^ mesenchymal cells in the dorsal area between the epidermis and the cortex at E16.5 (Fig. 2B). Furthermore, the thickness of the mesenchymal tissue overlying the dorsal cortical midline was significantly increased in the mutant compared to control (Student's *t*-test, *P*<0.001) (Fig. 2B′). These results suggest that activation of ß-catenin signaling in the premigratory neural crest induces the expansion of MSCs.

### The initial expansion of mesenchymal cells is dependent on ß-catenin signaling

**Figure 3:**
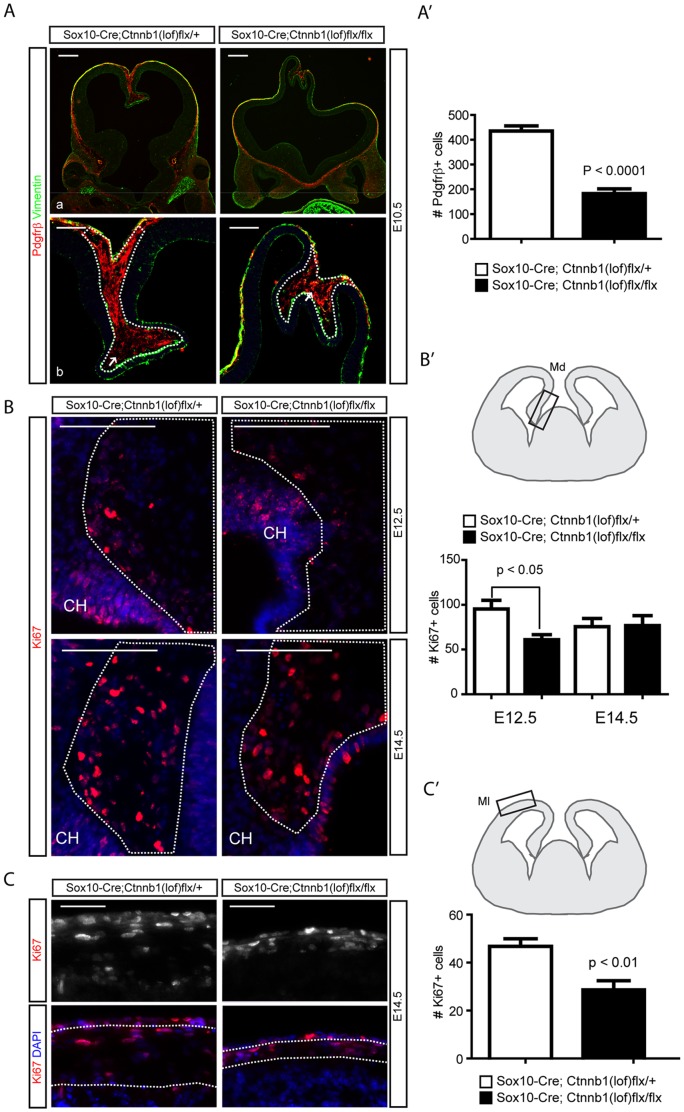
Normal expansion of neural crest-derived mesenchymal cells is affected by the loss of ß-catenin signaling. **A**) Immunofluorescence for mesenchymal cell markers Pdgfrß and Vimentin in Sox10-Cre;Ctnnb1(lof)flx/+ and Sox10-Cre;Ctnnb1(lof)flx/flx embryos at E10.5. Higher magnification images of **A-a** are shown in **A-b**. Arrows in **A-b** indicate mesenchymal cells in the dorsal midline bordered by the dashed lines. **A′**) Quantification of Pdgfrß^+^ mesenchymal cells in the interhemispheric region (n = 3). **B**) Ki67+ proliferating cells were counted from a region adjacent to the cortical hem (CH) at E12.5 (top) and E14.6 (bottom). **B′**) The drawing shows the area used to count Ki67+ cells in the dashed line and a graph represents quantification of Ki67+ cells (n = 3). **C**) Ki67+ cells were counted from mesenchymal tissues adjacent to the neocortex at E14.5. **C′**) The drawing shows the area used to count Ki67+ cells in the dashed line and a graph represents quantification of Ki67+ cells from the area (n = 3). Error bars indicate SEM. Md = dorsal mesenchyme, Ml = lateral mesenchyme. Scale bars = 100 µm.

While our results showed that activation of ß-catenin signaling was sufficient to drive expansion of the dorsal interhemispheric mesenchyme they did not necessarily establish the requirement for Wnt signaling in this process. To further examine this question, we used the *Sox10-Cre* line to conditionally inactivate ß-catenin in neural crest cells of *Ctnnb1(lof)flx* mice [10]. In *Sox10-Cre;Ctnnb1(lof)flx/flx* mutants, the telencephalon formed with an expanded, partially-invaginating, split dorsal midline at E10.5 (Fig. 3A). At E10.5 cells expressing the mesenchymal cell markers vimentin (Vim) and Pdgfrß [34], [35], were substantially reduced in the mutants compared to littermate controls. We quantified the difference for Pdgfrß^+^ mesenchymal cells and found a loss of approximately 50% in areas overlying the dorsomedial cortex (Student's *t*-test, *P*<0.0001, Fig. 3A′). To examine the proliferation mesenchymal cells in the dorsal interhemispheric region, we counted Ki67+ cells at E12.5 and E14.5 in mesenchymal tissues adjacent to the cortical hem. Mutant Ki67+ cells were significantly reduced at E12.5 and recovered at E14.5 (Student's t-test, *P*<0.05, Fig. 3B, B′). However, Ki67+ cells in the lateral mesenchymal tissues were reduced significantly at E14.5, perhaps resulting from the earlier loss of Ki67+ cells in the dorsal interhemispheric region (Student's t-test, *P*<0.01, Fig. 3C, C′). These data, along with our analysis of the *Sox10-Cre;Ctnnb1(gof)* mutants, supports the idea that ß-catenin signaling is necessary and sufficient for neural crest-derived mesenchymal cells to proliferate in the dorsal interhemispheric region.

**Figure 4:**
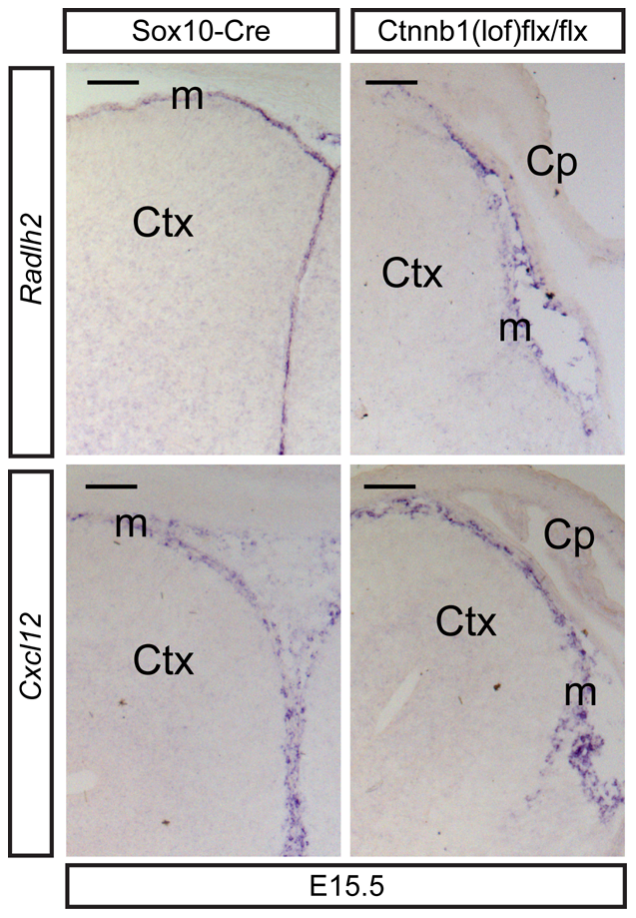
Conservation of the meninges in neural crest cells lacking β-catenin. Expression of meningeal markers, *Raldh2* and *Cxcl12*, in the embryonic midline at E15.5. Raldh2 was also expressed in the choroid plexus. Ctx = cortex, Cp = choroid plexus, m = meninges. Scale bars = 100 µm.

Next, we asked whether loss of ß-catenin in neural crest cells not only hinders the development of MSCs but expectedly also of cell lineages derived from them, such as the meninges. We recently reported that activation of Wnt signaling induces meningeal expansion during mid-gestation [36], which prompted us to examine by RNA *in situ* hybridization the expression of meningeal markers *Raldh2* and *Cxcl12* in the dorsal midline. Loss of ß-catenin expression in the neural crest lineages, however, did not inhibit the meningeal expression of *Cxcl12* (Fig. 4), as also previously shown for the selective deletion of ß-catenin in meningeal tissues [37]. Interestingly though, *Raldh2* showed a discontinuous pattern of expression, implying that ß-catenin signaling might be required for the expansion or development of *Raldh2^+^* meningeal cells. Consistent with these findings, we previously showed that loss of ß-catenin in meningeal tissues during corticogenesis affected the development of the pial layer and cells expressing *Raldh2* and *Bmp7* [37].

**Figure 5:**
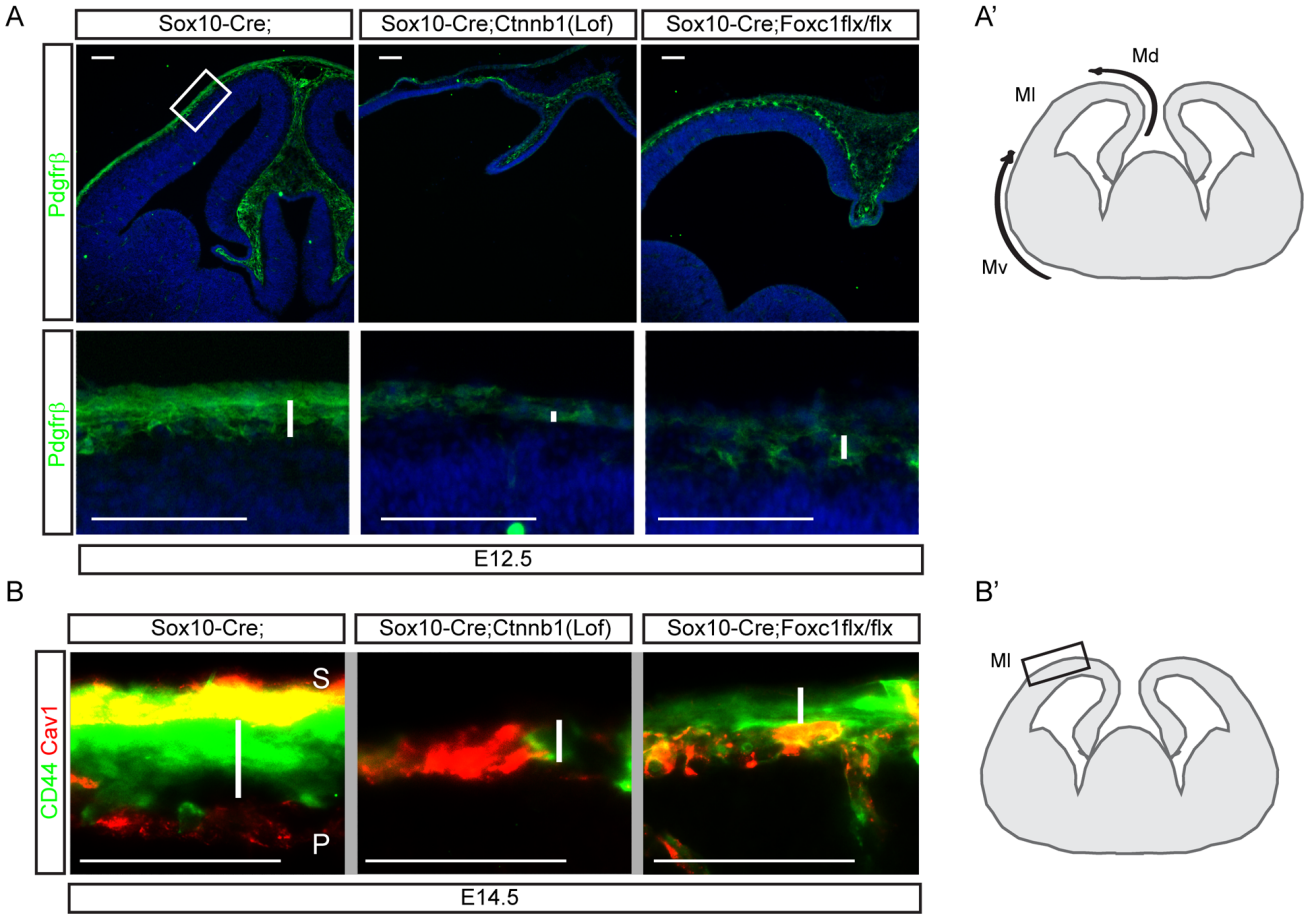
Failure of mesenchymal coverage of the neocortex after loss of ß-catenin signaling in neural crest. **A**) Sections of E12.5 embryos were stained for Pdgfrß. Dorsomedial Pdgfrß+ mesenchymal cells are shown in the top panel. Higher magnification images of neocortical mesenchymes are presented in the lower panel (corresponding to the boxed region). White bars indicate the distribution of Pdgfrß^+^ mesenchymal cells. **A′**) A schematic drawing shows two sources of migrating neural crest cells to the neocortex. Md = dorsal mesenchyme, Ml = lateral mesenchyme, Mv = ventral mesenchyme. **B**) Sections from E14.5 embryo heads were stained for CD44 and Cav1 to show MSCs and meningeal blood vessels, respectively. Thickness of the CD44 domain is reduced in both Sox10-Cre;Ctnnb1(lof)flx/flx and Sox10-Cre;Foxc1flx/flx mutants than the control (as marked by white bars). **B′**) A schematic drawing shows the region where images were taken (Ml). Scale bars = 100 µm.

We have previously shown that the Foxc1 transcription factor is critical for the development of the meninges [38]. To better understand the development of neural crest derivatives in *Sox10-Cre;Ctnnb1(lof)flx/flx*, we compared these to *Sox10-Cre;Foxc1flx/flx* mutant embryos, which exhibit a substantial impairment in meningeal integrity. At E12.5, the normal expansion of Pdgfrß^+^ mesenchymal cells was not seen in the dorsal midline of the *Sox10-Cre;Ctnnb1(lof)flx/flx* mutants. In contrast, *Sox10-Cre;Foxc1flx/flx* mutants exhibited a substantial number of Pdgfrß^+^ mesenchymal cells suggesting that the meninges are not necessary for the proliferation of neural crest-derived mesenchymal cells in the dorsal interhemispheric region (Fig. 5A). Pdgfrß^+^ mesenchymal cells covering lateral aspects of the neocortex were lost in both *Sox10-Cre;Ctnnb1(lof)flx/flx* and *Sox10-Cre;Foxc1flx/flx* mutants. This interesting result suggests that the meninges may be involved in the lateral dispersion of mesenchymal cells that originate dorsomedially, but are not required for the initial expansion of this cell population. At E14.5, when dermal mesenchymal cells become positive for CD44, a mesenchymal stem cell marker we further analyzed distribution of these cells [35]. Our analysis revealed that both mutant lines *Sox10-Cre;Ctnnb1(lof)flx/flx* and *Sox10-Cre;Foxc1flx/flx* exhibited reduced MSCs between the skin and the cortex (Fig. 5B). Since *Sox10-Cre;Ctnnb1(lof)flx/flx* mutants still produce the meninges, the loss of MSCs stems likely from the failure of initial transition of neural crest cells into MSCs. In summary, the differences in mesenchymal cell expansion between *Foxc1* and *Ctnnb1* mutants suggests that β-catenin function is critical to derive MSCs from neural crest cells while the meninges are required for the distribution of MSCs over the developing cerebral cortex.

### Defective development of calvarial mesenchymal cells in neural crest lineages lacking ß-catenin

**Figure 6:**
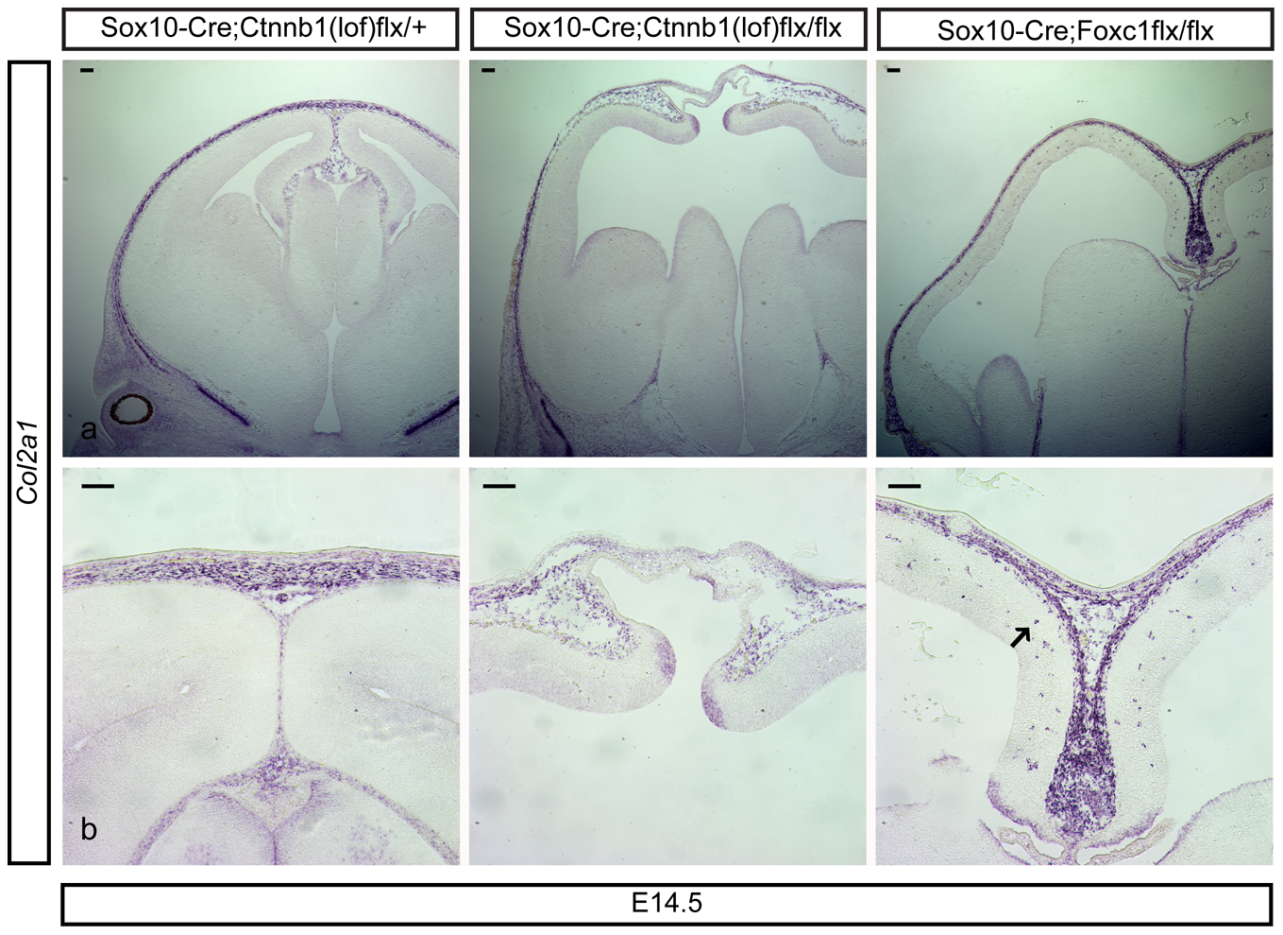
Defective development of calvarial mesenchymal cells by loss of β-catenin in neural crest cells. *In situ* hybridization of *Col2a1* was conducted to show the condensing calvarial mesenchymal cells at E14.5 (a). Bottom panels show mediodorsal *Col2a1* ^+^ mesenchymal cells (b). An arrow indicates infiltrating *Col2a1* ^+^ cells in the Sox10-Cre;Foxc1flx/flx mutants. Scale bars = 100 µm.

We showed that ß-catenin signaling in neural crest cells is critical for the initial proliferation of the interhemispheric fissure by MSCs. This finding raised the question as to whether inhibition of ß-catenin in mesenchymal cell precursors would further hinder the development of mesenchymal derivatives into dermal or calvarial cells. For instance, the majority of skull bones form by ossification of condensing calvarial mesenchymal cells originating from neural crest cells [39], [40]. Using RNA *in situ* hybridization, we examined the expression of *Col2a1*, a marker of condensing calvarial mesenchymal cells [23], [33]. *Col2a1* staining was similar to CD44 expression and was markedly reduced in *Sox10-Cre;Ctnnb1(lof)flx/flx* throughout the developing calvarium, presumably the result of reduced condensation of mesenchymal cells from a reduced initial pool of mesenchymal progenitor cells (Fig. 6). Interestingly, upon close examination, we found that *Col2a1^+^* cells had infiltrated the cerebral cortex in the *Sox10-Cre;Foxc1flx/flx* mutants (arrow in Fig. 6). These *Col2a1^+^* cells were found adjacent to endothelial cells, possibly the result of breaches in leptomeningeal blood vessels or ectopic expression of *Col2a1* in a population of cortical perivascular cells (data not shown). Strongly stained *Col2a1^+^* mesenchymal cells were still present in medial aspects of the developing skull at the cortical midline of *Sox10-Cre;Foxc1flx/flx* mutants suggesting that the loss of cells laterally may be due to defective spreading of *Col2a1^+^* cells (Fig. 6). To examine the resultant effect of reduced *Col2a1* ^+^ calvarial mesenchymal cells in the mutants, we labeled osteoblast cells and calvarial bone by alkaline phosphatase reactivity and alizarin red staining respectively, which confirmed in both mutant lines a severe loss of osteoblasts and bones tissue (data not shown). Summarily, our findings identified an essential requirement of ß-catenin signaling for calvarial formation starting from the onset of neural crest specification [10]. In general, our results supported the notion that the initial expansion of mesenchymal progenitors by ß-catenin signaling affects the later condensation of calvarial mesenchymal cells.

### Ectopic production of smooth muscle cells by inhibition of ß-catenin in the neural crest lineages

**Figure 7:**
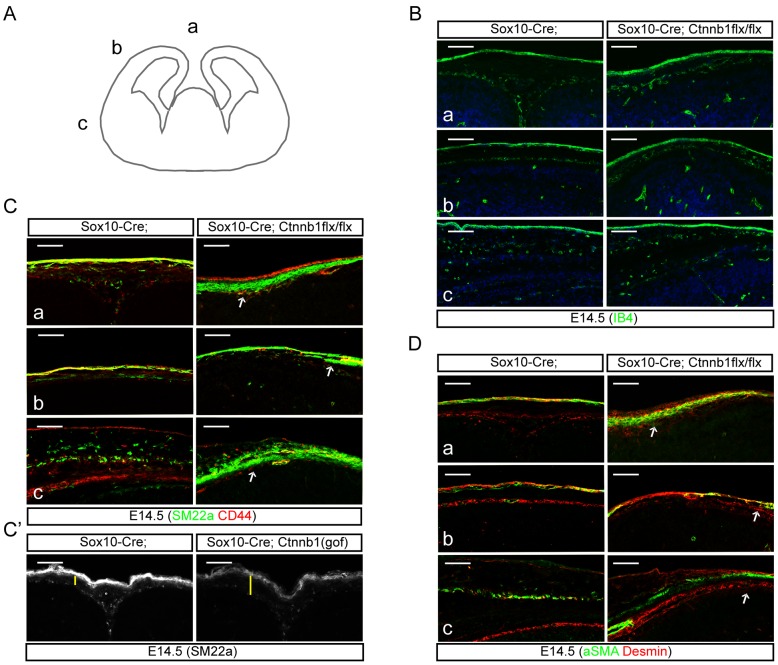
Ectopic generation of smooth muscle cells by loss of β-catenin in neural crest cells. **A**) A schematic drawing shows the three regions (a, b, and c) used to stain markers for smooth muscle cells. **B**) Isolectin IB4 staining shows distribution of blood vessels in the epidermis and meninges. Fewer mesenchymal cells were seen in the space between the blood vessels of Sox10-Cre;Ctnnb1(lof)flx/flx mutant embryos. **C**) SM22a, a marker for the smooth muscle cells, and CD44, a marker for MSCs, were used to characterize the mesenchymal cells in the regions a–c. **C′**) Sox10-Cre;Ctnnb1(gof) mutant embryos double-stained for SM22a and CD44. Yellow bars indicate the thickness of the mesenchyme. **D**) aSMA, a marker for smooth muscle cells, and Desmin, a marker for pericytes, were used to reveal the ectopic generation of smooth muscle cells from neural crest cells of Sox10-Cre;Ctnnb1(lof)flx/flx mutant embryos at E14.5. Arrows indicate the incompetent spreading of mesenchymal cells into the ‘b’ region of Sox10-Cre;Ctnnb1(lof)flx/flx mutants. Scale bars = 100 µm.

While considerable impairments in the number and distribution of MSCs were seen in the mesenchymal space overlying the developing forebrain, we wondered whether also mesenchymal derivatives that invade the brain and contribute to the vasculature of neuronal tissues may be similarly impaired. To further examine in *Sox10-Cre;Ctnnb1(lof)flx/flx* mutants the properties of residual perivascular derivatives derived from neural crest cells, we used isolectin IB4 immunofluorescence to identify blood vessels. As shown in Figure 7B, IB4^+^ blood vessels were present in the meninges and the dermis of the mutant with no tangible differences compared to control brains. To characterize remnant mesenchymal cells positioned between the meninges and the dermis of the *Sox10-Cre;Ctnnb1(lof)flx/flx* mutant, we tested markers of mesenchymal cells and found that markers for smooth muscle cells (such as aSMA and SM22a) were ectopically found in the mutants. SM22a staining showed a few dermal mesenchymal cells and smooth muscle cells in the control embryos, however, most neural crest-derived mesenchymal cells were stained for SM22a in the *Sox10-Cre;Ctnnb1(lof)flx/flx* mutant, while also losing expression of CD44, a mesenchymal stem cell marker (Fig. 7C). The interstitial mesenchymal cells in the lateral cortical ‘b’ area (see Fig. 7A) were presumably joined by neural crest-derived cells from the dorsal and ventral sources and were mostly absent in the *Sox10-Cre;Ctnnb1(lof)flx/flx* mutant. SM22a^+^ cells tapered off before reaching area ‘b’ likely indicating incompetent spreading of these cells (Fig. 7C, arrows). The *Sox10-Cre;Ctnnb1(gof)* mutant, however, showed an expanded dorsal mesenchymal layer and slightly reduced SM22a expression (Fig. 7C′). Consistent with SM22a expression, aSMA staining also showed ectopic smooth muscle cells after loss of β-catenin in the neural crest cells (Fig. 7D). Interestingly, the ectopic SM22a^+^ smooth muscle cells were not stained for desmin, a marker for pericytes suggesting these cells are non-perivascular myofibroblasts. These data suggest that loss of ß-catenin in telencephalic neural crest cells diverts neural crest-derived MSCs to become smooth muscle cells.

### Defective midline invagination after deletion of ß-catenin in neural crest lineages

**Figure 8:**
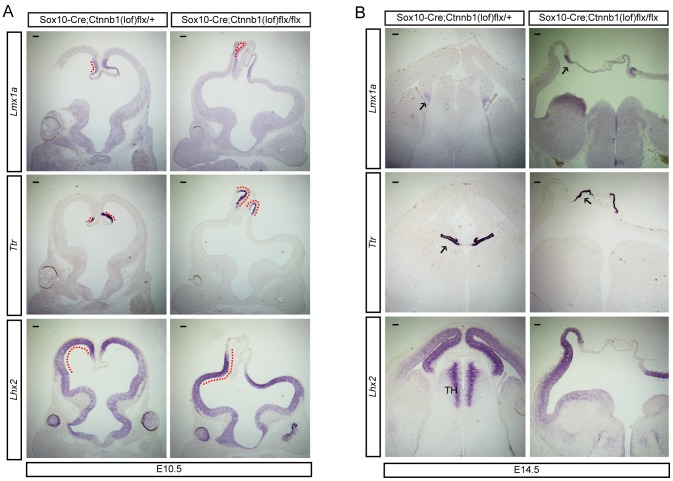
Failure of telencephalic midline invagination after loss of β-catenin in neural crest cells. **A**) *In situ* hybridization of midline markers. E10.5 embryos from Sox10-Cre;Ctnnb1(lof)flx/+ and Sox10-Cre;Ctnnb1(lof)flx/flx were used to show expression of *Lmx1a*, *Ttr*, and *Lhx2*. The dashed red lines highlight gene expression domains and the inverted dorsomedial telencephalon in Sox10-Cre;Ctnnb1(lof)flx/flx mutants. **B**) E14.5 embryos from Sox10-Cre;Ctnnb1(lof)flx/+ and Sox10-Cre;Ctnnb1(lof)flx/flx were used to examine the expression of midline markers, *Lmx1a*, *Ttr*, *Lhx2*. Arrows indicate the area of gene expression and highlight the failure of dorsal midline invagination in Sox10-Cre;Ctnnb1(lof)flx/flx mutants. Scale bars = 100 µm.

A most noticeable brain phenotype of ß-catenin inhibition in the neural crest lineages was the lack of the dorsal midline invagination (Fig. 3A) which was not previously reported in mice, which underwent *Wnt1-Cre* mediated deletion of ß-catenin, possibly because of even earlier loss of ß-catenin during neural crest development resulting in an abnormal forebrain and complete loss of craniofacial structures [10]. Further examination of cortical specification by RNA *in situ* hybridization using probes for *Lhx2, Lmx1a*, and *Ttr*, markers for the neocortex, the cortical hem, and the choroid plexus respectively, confirmed the expansion of the telencephalic dorsal midline including the choroid plexus in *Sox10-Cre;Ctnnb1(lof)flx/flx* mutants compared to control embryos (Fig. 8A). Interestingly, the neocortex of *Sox10-Cre;Ctnnb1(lof)flx/flx* mutants showed a reversed, concave curvature, presumably reflecting improper dorsal midline invagination (Fig. 8A; red dashed lines). We examined the development of the cortical hem, the choroid plexus, and the cortex at E14.5 by expression analysis of *Lmx1a*, *Ttr*, and *Lhx2* respectively. The *Lmx1a^+^* cortical hem was present irrespective of genotype, intriguingly though, in the *Sox10-Cre;Ctnnb1(lof)flx/flx* mutants the choroid plexus was exposed dorsally adjacent to the epidermis, which might cause the hypomorphic choroid plexus at this age. The massive cortical dysplasia evidenced by failure of midline invagination and lateral ventricle formation did not alter Lhx2 expression though, which was still present throughout the neocortical neuroepithelium in the mutant (Fig. 8B). In summary, our findings suggest that ß-catenin signaling of interhemispheric mesenchymal cells is required for the development and invagination of the dorsal telencephalic midline, and formation of the lateral ventricles.

## Discussion

In this study, we examined both loss and gain of function mutations of ß-catenin signaling in rostral neural crest-derived cells and showed that ß-catenin signaling is important for the initial expansion of MSCs in the dorsal interhemispheric region and the invagination of the dorsal telencephalic region. Cranial neural crest cells delaminate, migrate, and ensheathe the rostral cortex coinciding with dorsal telencephalic tissue growth and midline formation [1]. Wnt1 and Wnt3a are expressed in the dorsal midline of the neural tube and *Wnt1;Wnt3a* compound mutants show thinning of the dorsal midline in the hindbrain [9], however, the cortical hem and non-neural ectoderm are the major sources of Wnts near the dorsal forebrain [36]. The developmental period of cortical hem formation coincides with the development of craniofacial structures in the sense that Wnt signaling activates the expansion of neural crest derived mesenchymal progenitors thus enabling the supply of diverse mesenchymal cells to the developing face and head. Interestingly, the area of initial mesenchymal expansion is positioned adjacent to the cortical hem, a rich source of developmentally secreted Wnt molecules. The timing of these developmental programs led us to suspect that Wnt-mediated interactions of neuroepithelial cells and nearby neural crest-derived mesenchymal cells may occur. Consistent with this idea other mutant mouse lines with diminished cortical hems (such as *Foxg1-Cre;Ctnnb1(lof)*, *Nestin-Cre;Ctnnb1(lof)*, *Emx1−/−;Emx2−/−*, *Gli3*(*Xt/Xt*), *ShhN* and *Gdf7-DTA* mice) commonly show alterations in the positioning of the dorsal telencephalon with severe midline invagination defects [13], [15], [16], [17], [18], [19], [20]. Thus, it is possible that compromised invagination of the dorsal midline observed in those mutant lines stems from defective Wnt signaling in dorsal mesenchymal cells due in part to compromised cortical hem development or cell autonomous inhibition of ß-catenin function in neural crest cells. Further supporting this idea, our data here show that loss of ß-catenin expression in cranial neural crest cells leads to the failure of medial telencephalic invagination, a process required for the medial cortical area to expand and form the lateral ventricles. Moreover, previous studies have shown that removal of the roof plate causes patterning defects in the dorsal cortex [19], which lends additional support to the concept that there are important interactions between the cortical hem and interstitial mesenchymal cells.

As development proceeds, the derivatives of the roof plate such as the cortical hem and the choroid plexus invaginate and neural crest cell-derived mesenchymal cells fill the space between the ectoderm and the neural epithelium, which is required for the establishment of the ventricles. Formation of the lateral ventricles is essential to proper cortical development and function [41] and as our data show can be compromised by the loss of ß-catenin signaling in neural crest-derived dorsal mesenchymal cells without loss of meningeal tissues. Even though it remains to be further elucidated how ß-catenin signaling in mesenchymal cells triggers the invagination of the dorsal telencephalon, mesenchymal Wnt/ß-catenin activation appears essential.

Cells of the cranial neural crest contribute to diverse tissues of the head and face making neural crest-derived MSCs valuable for prospective stem cell therapies of neural crest-centered pathological conditions, such as the neurocristopathies [1], [21], [22]. These disorders, not only manifest themselves with craniofacial defects but can also lead to defects in the brain vasculature stemming from dysfunctional pericytes and smooth muscle cells [24]. Consequently, the list of known neurocristopathies is likely to expand over coming years with the further elucidation of disease mechanisms caused by deficiencies in the blood-brain barrier function of neural crest-derived pericytes [42]. However, the future demand for neural crest stem cell-based therapies will be severely limited by the small amount of available neural crest cell-derived adult stem cells. Consequently, understanding the molecular events integral to neural crest specification and differentiation of derived tissues is paramount to the development of any stem cell-based therapies. In this study, inhibition of ß-catenin expression in neural crest cells ectopically induced smooth muscles cells but hindered the formation of mesenchymal derivatives, such as dermal and calvarial mesenchymal cells, without severe defects in the formation of cortical pericytes and meningeal cells. Meningeal layers supply trophic and homing molecules for mesenchymal cells such as Cxcl12, retinoic acid and Bmps and thus the malformation of the meninges in Foxc1 mutants could lead to the inhibition of the mesenchymal derivatives [36], [38], [43], [44], [45]. The decrease of mesenchymal cells in the cortex of Foxc1 mutants is very likely secondary to the meningeal defects. The initial expansion of mesenchymal progenitors adjacent to the cortical hem in *Foxc1* mutants was not affected but instead spreading or targeting of *Col2a1* ^+^/Pdgfrß^+^ mesenchymal cells seems to be compromised. Future studies focusing on the interaction of meninges and non-meningeal mesenchymal cells will likely reveal meningeal factors controlling expansion and migration of mesenchymal cells and development of craniofacial tissues.

In summary, we show that ß-catenin signaling of neural crest cells is crucial for the neural crest-derived mesenchymal cells to expand and induces invagination of the dorsal telencephalon and formation of the lateral ventricles.

## Experimental Procedures

### Animals

All animal experiments were done with approval of the IACUC at UCSF. Mouse lines used in this study were previously described [*Bat-gal* [46], *Sox10-Cre* [47], *Ctnnb1(lof)* [10], *Ctnnb1(gof)* [32], *Foxc1flx* [48]]. *ROSA-lacZ* reporter mice were purchased from Jackson Laboratory. The day of vaginal plug was considered to be embryonic day (E)0.5. Mouse colonies were housed at the University of California, San Francisco, in accordance with UCSF IACUC guidelines.

### Immunostaining and *in situ* hybridization

Embryos were collected at noon of given developmental days, decapitated, and immersion-fixed overnight in 4% paraformaldehyde (PFA) in phosphate buffered saline (PBS). Harvested tissues were cyroprotected in 15% sucrose/PBS for 4–8 hours, embedded in OCT compound (TissueTek, Sacura), and frozen on dry ice. The frozen tissue was cut in a cryostat, mounted on glass slides, and sections of 12 µm were processed for immunostaining according to standard protocols while sections of 20 µm were processed for RNA *in situ* hybridization as previously described [36]. Primary antibodies used for the immunostainings were rat anti-Pdgfrα (Millipore, 1∶300), mouse anti-Vimentin (Millipore, 1∶1000), rabbit anti-Cav1 (Abcam, 1∶500), rat anti-CD44 (eBioscience, 1∶1000), rabbit anti-Ki67 (LabVision, 1∶200) and rabbit anti-Pdgfrß (Cell Signaling Technology). Isolectin IB4 Alexa Fluor conjugates were obtained from Invitrogen. Templates for RNA probes (*Lmx1a*, *Lhx2*, *Ttr*, *Raldh2*, *Cxcl12*, and *Col2a1*) used for *in situ* hybridization were designed according to the Allen Developing Mouse Brain Atlas. All comparisons in one experiment between control and mutant sections were performed on tissues stained on the same slides to account for variations between staining processes. X-gal staining in *ROSA-lacZ* or *Bat-gal* compound carriers was conducted as described (Siegenthaler et al., 2009). To stain alkaline phosphatase activities in osteoblast cells, 20 µm sections were applied to nitroblue tetrazolium/5-bromo-4-chloro-indolyl phosphate solutions (Roche) for 10 seconds. Confocal images were taken at the Nikon Imaging Center at UCSF using an upright Nikon C1 spectral confocal microscope and bright-field images were obtained using a microscope equipped with a QImaging CCD camera and QCapture Pro Software.

### Measurement of the mesenchymal cell number and thickness

For the counting of Pdgfrβ+ cells in the dorsal hemispheric region, three littermates were collected in 4% PFA for 1 hour followed by incubation of samples in 10% sucrose/1× PBS for additional 2 hours to prevent shrinkage of mesenchymal tissues. Twenty µm sections were stained for Pdgfrβ and Vimentin and one section at the level of the eyes was chosen to count cells based on the Pdgfrβ+ individual cells counterstained for DAPI (nucleus) (n = 3). Mutant sections were matched according to the level of the control telencephalon. To select the region for counting, a control section containing approximately four hundred cells in the dorsal hemispheric region was selected and marked the boundary as outlined in dotted line (Fig. 3A). Sections of mutant embryos were accordingly marked based on the littermate control and cells were counted, presented as a graph. Ki67+ cells were counted from the dorsal telencephalic hemisphere adjacent to the cortical hem (170 µm×220 µm) and the cells of dorso-lateral mesenchymal areas (350 µm×250 µm) were counted at the level of the cortical hem (n = 3). To show the thickness of the mesenchymal layer, tissues were fixed less than 4 hours and incubated in 10% sucrose for 2 hours to prevent the shrinkage of tissues. One representative image from three experiments was presented in each figure (n = 3).

### Statistics

Student's *t*-test was used for the pair-wise analysis of samples (SigmaPlot program, Systat Software Inc.). Error bars depict SEM.
